# Legal and Ethical Issues Associated With Challenges in the Implementation of the Electronic Medical Record System and Its Current Laws in India

**DOI:** 10.7759/cureus.56518

**Published:** 2024-03-20

**Authors:** Venkatesh Janarthanan, Senthil Kumaran M., Ninad V Nagrale, O. Gambhir Singh, Karthi Vignesh Raj

**Affiliations:** 1 Forensic Medicine, All India Institute of Medical Sciences, Kalyani, Kolkata, IND; 2 Forensic Medicine and Toxicology, All India Institute of Medical Sciences, Madurai, Madurai, IND; 3 Forensic Medicine, All India Institute of Medical Sciences, Guwahati, Guwahati, IND

**Keywords:** principle of ethics, challenges in implementation, legal and ethical issues, medical record systems, electronic medical record

## Abstract

Electronic health records (EHR) have revolutionized healthcare by providing efficient access to patient information, but their implementation poses various challenges. This paper examines the ethical and legal issues surrounding EHR adoption, particularly focusing on the healthcare landscape in India. Ethical considerations, including patient autonomy, confidentiality, beneficence, and justice, must guide EHR implementation to protect patient rights and privacy. Legal issues such as medical errors, malpractice, data breaches, and billing inaccuracies underscore the importance of robust policies and security measures. Threats to EHRs, such as phishing attacks, malware, encryption vulnerabilities, and insider threats, emphasize the need for comprehensive cybersecurity strategies. Overcoming challenges in EHR implementation requires meticulous planning, financial investment, staff training, and stakeholder support. Despite the complexities involved, the benefits of EHR adoption in improving patient care and operational efficiency justify the efforts required to address legal, ethical, and technical concerns. Embracing EHRs while mitigating associated risks is essential for delivering high-quality healthcare in the digital age.

## Introduction and background

Medical records are the document details that clarify the medical history of the patient, clinical findings, laboratory investigation reports, surgical operative notes, patient's condition, and past and present prescription records [1]. According to the American Health Information Management Association (AHIMA), the digital form of medical records is classified into electronic medical records (EMRs), electronic health records (EHRs), and personal health records (PHRs) [2]. EMR is a digital content of charts containing patient registration data in outpatient and inpatient departments, billing, appointment, and scheduling for checkups/follow-ups, monitoring the quality of care to patients. The main functions of EMR are the electronic entry of data, preservation, and safekeeping of digital data. EMR is a part of EHR and is mostly compatible with each other. EHR is a longitudinal electronic record of data about the health of a patient created by physicians in any healthcare setting. EHR comprises the treatment record of the patient, demographics, test reports, present and past illness, and medical history. PHR is the gathering of vital information about the patient's health or the health of family members, such as a child or parent, which they actively keep and bring up to date. Although the initial period's medical records should be kept on paper with advancements in technology, it is essential to change from a paper medical record to a digital form of preserving the health record [2].

The main advantage is making patient information more readily available to many people than any paper chart stashed away in a record room. However, with this increase in recent technology, there is a rise in e-legal risks like breaches of confidentiality and privacy. A ransomware attack that happened in the premier medical institute in India in the recent past hampered the hospital's electronic record system, which caused difficulty in delivering health care to patients for several days and allegations regarding software program manipulation. These issues are increasing day-to-day in routine courtroom proceedings [3-4]. The foremost challenges in implementing the EMR are unsatisfactory planning, deficient interoperability, limitations of technical resources, data migration (paper to electronic record) leading to doubling the work of staff, workflow interruption, fewer human resources, and no affordable software cost [5,6]. This paper mainly highlights principles of medical ethics sticking to EMR, legal and ethical issues, chances and prevention of occurrence of malware attacks, challenges and their resolutions in implementing EMR, guidelines, and related laws in India, which will help adopt the EMR system for delivering the utmost level of healthcare to the patient at hospitals.

## Review

Ethical issues and principles of medical ethics related to EHR/EMR

Autonomy

The patient has the right to decide whether to register an EMR regardless of what the hospital authority believes is advantageous to that patient. Suppose the patient refuses to register into the EMR and proceeds to healthcare facilities. In that case, the hospital authority can still attempt to inform patients about its benefits and the consequences of not opting for it, like the chances of missing case sheets and medical records. Patient autonomy may be affected by family members' influence or compulsion of any other staff, and hospital authority can proceed without permission on behalf of the patient. However, the healthcare providers can proceed further for registration into the EMR without any consent from the patient for good faith in saving lives during an emergency [4].

*Confidentiality* 

The patient believes the physician/hospital authority that information about the illness or health record and condition will not be permitted to be revealed to others. Suppose information leaks that may harm the patient or be taken advantage of by others; the unauthorized access, disclosure, and secondary use of patients' information deprived of their consent or against their wish will violate the patient's secrecy. Therefore, access can be limited to viewing patients' health records so that modifying or deleting the content of data entered by healthcare professionals can be avoided. Certain exceptional circumstances, including notifiable infectious diseases, such as COVID-19, cholera, and HIV/AIDS, should be informed/shared with concerned health authorities/others without consent from the patient [5].

Beneficence and Non-maleficence

Beneficence (duty to do good) justifies clinical and biomedical research using EMR data that will benefit individual patients and society. However, it conflicts with non-maleficence (duty to avoid harm) if the health information and patient identity are revealed to the public, which may harm the patient's dignity. Thus, information about the patient's identity and data must be kept trustworthy and not be revealed at any point during or after the research [6].

Justice 

The patient who refuses EMR/EHR might be at a drawback of not getting proper quality healthcare service. This can be avoided by giving patients awareness about the advantages of EMR/EHR and obtaining informed written consent from patients about maintaining confidentiality and privacy of patient information before collecting the information from the patient. This principle states that all patients will be treated equally. It means respecting the patient's rights and treating them equally, irrespective of whether others agree with this EHR system [7].

Legal issues with the EHR/EMR

Chance of Medical Errors

During the collection and documentation of data, staff may copy and paste notes between patients’ data, which can reduce the truthfulness of the medical record. EHR/EMR systems do not generate a template for each clinical condition. Templates that are narrow in scope or do not permit information entry or impressions signifying different diagnoses can expose the employer to legal issues. The erroneous documentation of the name, age, gender, clinical diagnosis, and concerned treating doctor on the patient’s record or medicolegal case documents. 

Errors in the EMR may lead to the patient’s health/law enforcement agencies being at risk if they are not corrected immediately. If it's a paper record, there's a chance of file deletion, or errors in documentation can be corrected with a single-line strike-through. Subsequently, the entry should be signed and dated. In the case of EMR/EHR, making changes to the record after saving the data and rendering it not editable later becomes very difficult. It is advised to issue a separate corrigendum regarding correction in personal data stating spelling correction or an error related to wrong entries by concerned signatory staff or authority [6,7].

Possibility of Medical Malpractice 

Switching from a familiar paper record system to a new EMR system can introduce the possibility of medical malpractice, such as inappropriate treatment of patients without analyzing medical records due to potential mistakes during the transition, which can adversely affect medical practitioners. EMRs significantly impact litigation related to medical malpractice due to the readily accessible documentary evidence they provide. This evidence can protect a practitioner from false malpractice claims or serve as proof for civil/criminal trials in a court of law. Clinicians heavily rely on EMR resources such as diagnostic results, treatment records, investigation reports, and reminders for follow-up care. However, if the EMR system experiences shutdowns or disruptions, clinicians may encounter significant challenges in accessing critical patient information, potentially leading to complications in patient care and legal ramifications [7].

Data Breaches

EMR is the most targeted in healthcare breaches. The risk of breaches affects the security or privacy of patient data, the possibility of hacking, destroying the data, incorrect paper to electronic transmission, and error in treating patients. According to the Data Personal and Data Protection Act of 2023, illegal access to secured health information in India results in high penalties (Table 1).

**Table 1:** The purpose of various laws and recent acts related to electronic medical records.

Current laws and acts	Specifies/Purpose
Digital Personal Data Protection Act, 2023 [8]	India’s first privacy act aims to safeguard the digital personal information data of citizens including the right to privacy to person and upholding the responsible data management practices. This act affirms and regulates individual privacy rights and corporate company’s real data processing neediness without breaching data privacy. The breaching of the data privacy of a person leads to fine and Imprisonment.
Article 21 of the Constitution of India	It lays down (the right to Privacy) the fundamental right of a person [9]. This was acknowledged by a nine-judge bench of the Supreme Court judgment by Justice K.S. Puttaswamy vs. Union of India, directed to creativities by the government towards the formation of the sensitive personal information protection laws in the national health policy, 2017.
Information Technology Amendment Act (IT Act), 2008	Section 43A- If any corporate company fails to maintain the sensitive personal data or information, that causes wrongful loss or wrongful gain to any person, then such corporate shall be liable to pay damages to the affected person. Section 72A- deals with punishment for the revelation of personal information/data in breach of lawful contract and any person may be punished with imprisonment for a term not exceeding three years, or with a fine not exceeding up to five lakh rupees, or with both. Shortcomings of Act: Restraint to corporate company’s responsibility towards automated processing of data. The clients are not able to take applications about their requirements. There is no provision in this act on data localization which was the major issue and reason behind the banning of the Chinese applications in India. To overcome these limitations in India, there is a neediness of data privacy law/Act [10].
Section 403 IPC	Defines criminal penalty for dishonest misappropriation or conversion of “movable property” for one’s own use, which shall be punished with imprisonment of either description for a term that may extend to two years, or with fine, or with both.“Movable Property” considered to be the transfer of Digital personal data information [11].

The hospital administration should make policies and plans to train the employees to comply with EHR standard guidelines by the Ministry of Health and Welfare, Government of India. In aiming for minimal EHR costs and embracing modular standards rather than standalone ones, it's essential to prioritize patient privacy. This involves obtaining authorization from the patient, while trust is established through a reliable third party verifying identity. Protection of sensitive personal data, including passwords, financial information, and biometric data, is fundamental. Records should be retained for the duration of a person's life and up to three years following their demise [12]. Psychotherapy notes obtained from an anonymous source with assured confidentiality are treated with the utmost care. Access to recorded patient/medical data should only be granted to healthcare providers upon request, ensuring confidentiality is maintained effectively [12]. The incident related to the violation of rules and policies can be reported timely, and an immediate action plan should be executed to resolve the issues. The documentation of such incidents should be preserved for future purposes if an inquiry happens related to violations used as documentary evidence.

Wrong Billing Claims

EMR systems have opportunities for wrong billing claims and are subject to strict monitoring and scrutiny with proper records [12,13].

Threats to EMRs and EHRs

The healthcare sector remains a significant target for data breaches, particularly during the COVID-19 pandemic, where record-breaking costs were incurred. According to IBM Security, healthcare breaches carry the highest price, averaging $9.23 million per incident. A U.S. Department of Health and Human Services report highlights that approximately 578 healthcare institutions experienced data breaches, impacting over 41 million individuals, including institutions in India [14].

Phishing Attacks

Phishing attacks, a type of social engineering, involve perpetrators posing as trusted sources to deceive targets into clicking malicious links, opening emails, or divulging login credentials, leading to malware infiltration. Mitigating such attacks involves refraining from clicking suspicious links or opening anonymous messages/emails. Before proceeding, physicians should be educated to verify all requests for sharing EHR data [15].

Malware and Ransomware Attacks

Malware attacks on hospital EMR/EHR systems typically stem from unauthorized access, weak password management practices, disloyal insiders, lax physical security protocols, and theft of electronic devices containing health data. Refrain from constant warnings and recommendations by computer staff/operators exacerbates vulnerability. EHR systems require adequate time to review warnings and perform automatic file repairs to prevent corruption or deletion of patient data [16]. Malware may infiltrate healthcare system networks through various means, including phishing attacks, software vulnerabilities, downloads, and encrypted traffic, posing risks ranging from data theft to network and host computer damage. Ransomware, a form of malware, locks users out of their computer networks until a ransom is paid, jeopardizing access to up-to-date patient information in hospitals.

Encryption Blind Spots

Encryption of EMR/EHR data during transmission offers protection; however, encryption blind spots pose significant risks in IT healthcare, enabling threat actors or hackers to evade detection, conceal activities, and execute targeted attacks unnoticed [17,18].

Cloud Threats

Cloud storage presents vulnerabilities to hacker attacks. Effective mitigation strategies involve staying ahead of cloud threat actors, addressing common cloud security issues promptly, and understanding the implications and vulnerabilities associated with open-source software use in the cloud.

Insider Threats

Insider threats are prevalent across industries, including healthcare. Establishing and enforcing a comprehensible cybersecurity strategy and policy within healthcare organizations is essential. This involves educating all healthcare personnel, strengthening administrative controls, diligently monitoring physical and system access, implementing workstation usage policies, conducting audits, monitoring system users, employing device and media controls, and implementing data encryption measures [19,20].

Tips for navigating an EHR

Regularly updating your operating system and other software on time is essential to prevent potential cyber threats on your devices. The organization should strictly prohibit the use and distribution of pirated software. Therefore, it is mandatory to use only open-source or licensed software on computers or laptops connected to the organization's network. A botnet refers to a collection of compromised machines known as *bots*, which can be manipulated remotely by an attacker.

They utilize these compromised machines to execute Distributed Denial of Service (DDoS) attacks, distribute spam messages, infect additional machines, or conduct other malicious actions. The following precautions are recommended to mitigate such incidents. Regularly scan your computer with updated antivirus software and disinfect if necessary, maintain updated antivirus and anti-spyware programs, deploy and manage a personal desktop firewall, monitor for suspicious network activities and disinfect as needed, utilize only genuine software, ensure operating system and application software are kept current with patches and fixes, be cautious when opening email attachments, avoid browsing untrusted websites or clicking unsolicited email links, refrain from sharing passwords, and avoid using untrusted WiFi or hotspots [21,22].

In a hospital setting, EMR policy should be clearly defined, and standard operating procedures can be framed for using digital health information systems. The hospital staff should be trained on hospital policies, procedures, prevention from malware attacks, security and trustworthy requirements, and consequences of violation of EMR policy [23,24]. Hospital administration can opt for malpractice/disciplinary insurance for the anticipated increase in medical professional legal responsibility claims concomitant with the usage of EMR [25].

Challenges in implementing EMR

Inadequate Planning

Introducing an EHR brings about a cultural transformation within the organization beyond technological progress, constituting a substantial management shift. Effective planning for EHR implementation is crucial to mitigate risks such as data breaches and cybersecurity threats to patient information. To address these challenges, all stakeholders must be fully dedicated to strategically executing the implementation of EHR systems with meticulous planning.

Implementation Cost

The utilization and implementation of health information technology, such as EHRs, can present significant financial challenges, particularly for minor healthcare practices. The expenses associated with acquiring and deploying these technologies, including personnel, training, infrastructure, and ongoing support can pose hurdles due to limited resources.

Time-Consuming

Healthcare providers and their teams may need help to allocate the necessary time, effort, and resources for staff training throughout the EHR implementation process. This challenge could impede EHR adoption during the training stage. The solution lies in introducing a comprehensive training regimen for staff members, emphasizing the benefits of the new system in enhancing patient care while simplifying and boosting their productivity in their roles.

*Staff Limitation* 

The acceptance of EHRs by patients and providers may fluctuate over time, with some potentially abandoning them prematurely due to initial technical glitches. There are instances where staff members may need to be educated and trained about the latest technological advancements and the numerous benefits of EHR implementation. This delay can hinder the timely adoption of EHR systems.

Workflow Disruption

Regrettably, if the implementation of an EHR is not appropriately customized to fulfill its intended role, it can sometimes disrupt the workflow of practice entirely. Furthermore, this disruption may stem from insufficient demonstrations by your vendor regarding how the implementation will operate within your specific practice.

Privacy Concerns

When utilizing EHRs, certain healthcare professionals, and patients may harbor apprehensions regarding privacy. A prevalent worry involves the potential loss of medical records due to cyberattacks or breaches.

Data Migration

Transitioning paper-based records to digital format poses a significant logistical challenge for staff members. Managing numerous documents containing the medical histories of multiple patients makes data entry an uphill task, amplifying the challenges associated with EHR implementation for hospitals [26].

## Conclusions

EHRs present significant benefits and challenges for healthcare organizations. EHRs offer enhanced accessibility to patient information and improved efficiency in healthcare delivery. However, transitioning from paper-based records to digital formats requires careful planning, resource investment, and addressing various legal, ethical, and technical considerations. Ethical principles, including autonomy, confidentiality, beneficence, and justice, must guide the implementation and use of EHRs to ensure patient privacy, autonomy, and equitable access to healthcare services. Legal issues such as medical errors, malpractice, data breaches, and wrong billing claims underscore the importance of robust policies, training, and security measures to protect patient information and mitigate legal risks. Threats to EMRs and EHRs, such as phishing attacks, malware, ransomware, encryption blind spots, cloud threats, and insider threats, highlight the need for comprehensive cybersecurity strategies and continuous monitoring to safeguard sensitive health data. Navigating the challenges in implementing EHRs requires adequate planning, financial resources, staff training, and stakeholder support. Despite the complexities involved, the benefits of EHR adoption in improving patient care and operational efficiency justify the efforts required to overcome these challenges. In conclusion, embracing EHRs while addressing legal, ethical, and technical concerns is essential for healthcare organizations to enhance patient outcomes and maintain the integrity and security of health information in the digital age.
